# Supplementary material for: CRISPR/Cas9-Based Mutagenesis of Starch Biosynthetic Genes in Sweet Potato (Ipomoea Batatas) for the Improvement of Starch Quality
Source: Int J Mol Sci. 2019 Sep 23;20(19):4702. doi: 10.3390/ijms20194702 (PMC6801948; doi:10.3390/ijms20194702)
Supplement: Supplementary file 1 [file ijms-20-04702-s001.zip › ijms-583672-for proofreading sup/Supplementary 1 for resubmission 9 4 2019.doc]

IbGBSSI genomic sequence

atggcgactataactgcctcacactttgtttctcatgtctgtgggggtgccacttctggagaatcaaaagtggggttgggtcaattagccctgaggagccaagctgtgactcacaatgggttgagacctgtgaacaagattgacatgttacaattgagaacaagtgccaagaaacctagcaaaaatggaaggaaaaatgagggtggaatggcagcaggaactattgtgtgtaaacaacaagggatgaacttggtctttgtggggtgtgaggtgggtccctggtgcaaaactggtggacttggagatgttcttggaggattgccnnnnnnnnnnnnnnnnnnnnnnnnnnnnnnnnnnnnnnnnnnnnnnnnnnnnnnnnnnnnnnnnnnnnnnnnnnnnnnnnnnnnnnnnnnnnnnnnnnnnnnnnnnnnnnnnnnnnnnnnnnnnnnnnnnnnnnnnnnnnnnnnnnnnnnnnnnttcagtggaccttatggtatttttcttcttctagaggttttggaagtacttgagattagagtgtggaaagaattaacataataatcttttatgaaattgtaaaggtgaggatgttgttttcgttgccaatgattggcacactgctctccttccatgctatctgaaaaccatgtaccagtcgagaggaatctacatgaacgccaaggtaatgcctcttttggattggactggacggaagttttccacatttaatcctacctaatcacaaggactatacttatatgtgttcccgctatttgctaaaatgttgccaggttgctttctgcattcacaacattgcctaccaaggcagattcgccttttcagacttttctcttctgaatctgcctgacgagtacaagggttcttttgattttattgatgggtaggaattagatgcttttagctaaggttatgaattatgtatcttgattcttgctgcttgatctctgaattcaattcattctcaatattgccaggtatgacaagccagtgaaggggagaaagataaactggatgaaagctggaatccgtgaagcagacagggtttttactgtgagcccaaactacgccaaggagcttgtttcttgcgtttcgaagggtgtggaattggacaaccacatccgagactgtggcatcacgggtatttgtaatggaatggatacccaagagtggaaccctgcaactgacaagtaccttgctgttaaatatgatatcacaactgtaagattagtactttgtgtctacattctcatttgatattgaatttagggagttttgctcattcggaattttatgctttataggttatgcaagcgaagcccttgttgaaggaagctcttcaagctgcagttggtttgccggttgacaggaatattccgttgattggttttatcggcagacttgaagagcagaaaggctcagacattctttatgctgcgatttctaagttcatttcaatggatgttcagatattgattcttgtaagtgctatgtggactcttaatgcatgcttttcttacgtccgtccctttgccaaaccatggatctgaattgcaatgatgaaatcaatcagggaactggaaagaagaaatttgagcagcagattgagcagcttgaagtgatgtatccagataaagctagaggagtggcgaagttcaacgttcctttggctcacatgattactgctggcgctgattttatgttgatcccgagcagatttgaaccgtgtggtctcattcagttgcatgctatgcgatatggaacagtaagaaacccgacacacttaaacctatccaaaactctcctttcatgattcttcctagttaaagttctaattgtcgatataaatgtgtacatatatcagccgtgcatctgtgcctcaactggcggactcgttgacactgtgaaagaaggttatacagggttccacatgggggctttcaatgtcgacgtatgtgattctcagcataatacattctttttgtctattaattatgttatatatcataacaagaggtaacgaagttttgttgttgcttgtgctcagtgtgaaactgttgacccagaggacgtgctgaaggtgataaccactgttggtagagcacttgcaatttacggaacccttgcattcactgaaatgatcaagaactgcatgtcacaagagctctcgtggaaggtaggcattcgcccttgttgagcataaatataatataattcgcccttgttgagcataaatataatatataaacgaaaatgtgcagtccaactatcagcttatgcttttagttgagatggagcacatgcttcaatttggtatcagagccatgcaaaaagtcatggttcgaatctcccgtgttgagcataaatgatatataaacgtttgccctaccgggttcaaataacagttcttccttgtgtacagtttcaaagccgctaactggttttcgttgtagggacctgccaagaattgggaaacagtgttgttgagcttaggagttgctgggagcgagcccggggttgaaggagaggaaattgcaccgcttgcaaaggaaaacgtcgctactccatgaaaactatggtctcaaaacctacaataagctggcatatgaaactaaagttttatatatgttgaaagagcggaggcgttttggtgctcttgagcattatgaggtgatgaaagagtcaatgggggactctttctttattgttttttggtttgcccaggtggtagtaaatagagagttcaaactaactagtttggtgttttaagacattgggaacttgcacttcagcaggctaaatgtataatataaataaacaatagcctcttgtggttttgtgatttgatttccagcaacttttgatgattttgttatattatatatatatatatatatatatatatatatatgaattcattctctacctttagcacactgcatttagaatgctgtcaaaatgctgatagttaagtcatagcattcattaggtatttctagaatctgaatgcggctggcaatcccacttctcttgggataaagttgtctttagtttctttttaaagggcgtggagcagtgcacttttctggaattttctgctagactgagcacttttcttgggacggagtggtggagttttgtctcattcgaagacaaaatatgttgttattttacatcaagctccttgctcaggcttatgttttaccatcagatttatttatggactatgcttctcatggatgagtttcttttgggctgcaatatatatattttccgactctcaatcagtgggccatcacaaagactcacacatgggctggaattgatccagctagtctatttttaagtttgatacagccttgaaggcattggtttatgttttagatttgggcctacgcctagatttgtactcaggggtaaatgccccggtgtttcatgtat

IbSBEII genomic sequence (10491bp)

atggtttacaccctctccggcctccgattgcccgctgtcgcgccggtttacaagcatatcggatcgacctcgcacgccgatcggcggaatgccaatcccactctctctctgaaaaggaactcgttttcttcatgtatgtgtgtgtttcctgggttggattctgatccgagattctatgcctttatcgctttatgagaccaaaaaggcaatggccgaaaagcgaatcttttgaatgcttttaggagctttgattattcgtagatttatcgtttttgattcaaatttgatatcatcgtgctgcgtgcgatcaatgtgtagttttagtttagaaacagcgggactgttagctttgccttttaatctttttgttgccttttagtaaaaaatatatactgttttcaaaaaaaaggaaaaaatatatacttgctgcctagctcaatttagagaataatgatgaaagctgagagcttttctcaaaagcgatcgtctttcaactatttatttaattgcatagattcgattctctttgtttactgacactgaatattttatattttctgctatgctggtggattagagatttttagtggcaatgctatttaaaaaaacaattaagttaatctatctttaatatctggaatctccattacttagtttggtagtaatcaaaatcttctggatatgttttactctgtacctttctgtttgataacaacacccatactatgactaatttgcaggaatgacttttcttatcagttagttatatcttttggacctaatttgctttgcttgatatttccaagaaacttaaccagtttggtgcttggctgcagggaagatctttgccggaaatacttcctatgaatccgaaccctcttcttttagagttgcagcatcagagaaggtccttgttcctggcggcgagggcgagggatcatcgtttccaactgatcagttagaggttgctgaggcattatcagaagatacccaagtatgtgtatgtgtaaacctactgtcatagaacatgagcacaagactctttctcttaaataaaagattattactcatttaaaataaaaagtaaaagttataactgtgaattaaaattaattattcttcagtatttacgaatatcaattactaaataagtttgcagatagatattctttataaagaatttgtgaataaaaattatgagttatttctttaacatttgctatttatgaagatataaattgcaacaaaaagttgcaaataaatattcatgttaaacaagtctgaaatgatggtgataagttgcagtataaataatttggacagaaaaggtttttggcatctctcatgggatggtagcatgctactggcttggcaactagggctgggttccttgcaaatctagaaataatagcagtggcgaatctgcagttgttgcttgaagcagtaagatacttttaagaattaagatcatgtattttgtcaaaagatcataatacctacttatagaaaatgctatagggggttatgcctcatcattaacgagatgtgtcatagtgtctgtattgcttttaaaggcagctgcttagtaaggtggaagtcccaaaagccaatgcattatagttgaaagtgttattcttctaattactagaagaaatacgatatcttgaataactctccaaccatgccatattccccacaaaatgaccatggcataaaattgtgttaggttgcaatcttcatacatatgtggatcctttcttcagcatgattgtcttattattggcatgatttcttttatactcatgatcgtcttattaattctaatgtcgaggtactcttggagtcttggtttttgttcatcacattttcctgtaatatcacaattgttttcactcattgctatcttaggtatcggctgacgtggataatgtgaagatggaagagaacagcaacagtgaaagcaatgtggatttcgtcaaagcagcaagtgattctaaagaaagtgttcaagagcaggatcacacgtcttcactacaatttgaagaagatggtaatgtggaagtttcacaaaaaccagagactttggatgacatcagtgctgaatctgaaatggtgaaaaagagggccattcctccacctggacttggtcagaggatctatgagatagaccctctcttgaaaaattttcgtgaccatcttgattataggtaacacaccttcacaagtgtaaattctactagtgcaattcattgaactgcaaaatgagtgcaaatgatgtttgattatcactaggctgctgcattttgttcttacatcaatctgtcatgcatactaccatagcttagcactgtcctcaaaccactatatgttattcttatttttcagctgtatatttcctgtcttagcaactcttctctttgtttcagtactattattagtttcttttctttattttttttccagctgctaaaactatagctttacaatgccatgagttttatgaaacaattgtatcttcaacctcctcctccctgtgctccccctccacacacacacacacacacacacacaccccccacccccaaaaaaacaaaaaaaaagagatagaaacattccctttgaatttggcttgaaatagaactgcagctaactttaaaaatgccatgcctttacatctctgtatcaactatcaagttcttttcatgaatctaaagctctcatatctgttcataaagttcccatacctgaggaggttacctggatattaatttcttatttttatattttccaactataggttttcgcactataggaaaatccgggaagcaattaaccagtatgagggtggcttggaagtattctctcgtggctatgagaaattaggtttcactcggaggtaaacagcattgctgtcatattttttgctctgcacgtctacaattcttgtatgatgtagtagtgcactctgcacttttctttggctgcaaaagaaatgaacttaatagctatttcttaattaggtagtgcattgtattttactcaattgtggaatcttactttcagtgttgaactattaaatgaatttaaacaccagagcctaacctttaggaaggatcaggaaaaggtatggtataactaaaattaaagctttgactattagatataaggtgacccctcagcaattgtcaaattcatttctgttttattctactgtattacgagaaatatgcaatgctccagaagcctacttggtaaacttcatgtgaggagatatttccatgtaaatgtgcttaatgagatgattcttttgaaccttgaaattgatgtggtgggagtaaaatttgggtgggtggtagttgcagagtttatgttgtggctagcaaggccagatccattggtacttctgcatctaagaaaagagccaataccctagagaaaaggaaaagtaaaaagaaaaaagggagcctcgataaaataatggtcatcaatgtgctcatgataaccactcatcccctggcatgcaccatgcacacagagacaattatctctctctctctctctctctccctaaagattccctataaatgaattttgatgacgttgaataatttcttgctcattgcatgtgattttttttttttttactgccagtgctacaggtatcacctatcgggagtgggctcctggagccacggtcagtattgctctttttcattgctattgttggaatcaggttttcagttgttataagttcttcttttccctgcttttgtagtgggcaacactcattggagatttcaacaattggaatccaaatgctgatgttatgactcgggtatgcatttattggttcagttctgaatacttagttattctgtttgattggcatcttttggtgggagattgatacccttgctgtggtttagcttaattcttgatgctgatggtcttttattgcttcatctgcatgtttattagctcaatgcttcatatggacattagttggttggacatgttcaatgataaaaaacaatcaatttctttcgagttacatatttctttttatggtttacccatccacatgttaatagttggtctgttttgtttcccaattttgatttgattaggcactctgggaagctattatgaaataagaattaaataatcagttaattactcaatttactgttcattttaaagggattaaaacttgatcaatttaattgcattatctcttctatctaggtatgctagactttgtaggaagccaaatttccttctgtaagtatggctggggacctggggttaacctgttcaagcaaaggtgcaatattacatagattagtgaattagaaaaatgtgtgagcttgcatttatttacattagttagtgattacaatgagtccatagaaaaatgtgtgaacttgcatttatttacattagttagtaattacaatgagtccatgctacattataatcatttctagcttttaaacagaatgaatttggtgtttgggagatctctttgccaaacaatgcagatggctcacctgcaattccacatggctctagagtgaaggtattggactatttgatggttgtgctattgaaatttgttcaacttgttgtaaaggaagctgtagttttgaaagaatgaaagataatgctaatatgaatgaaacaaaggtcttatcccaaatccttgatagctgctgactgcaaattctatcaagtatatatcacaccacaatgctcacacatgctttctatctttctaattcagacaacaggctctcttcatataaaactcaaccccaccccacataggtccatgttattttctttttcttacatttttttgtatgctttattattttgtattggaattgcagatacgtatggacactccttcaggcattaaagactctattcccgcttggatcaatttctctgtacaagctcttggtgcaattccttatgatggaatatactatgatcctccagaagaggtattcatttttattctattgctgcaaaaacagtcaatgcttaaatgttgagatcttctaaataaggctggagacaagaaatgaccaatgattctttgtaatttactcataagacaaatatgcttagatacccttgtatgatctctttccttctcctgttctgctgttaaaactctcatgtttcctttagattactaatttgtgaattagacgatagtactttgtactggtattagccgggcatgattcttttcttcctacgaagttaagatgtttgtgttatttatcacatcaacaaaaggatatattttctttcaatttcttctgttgcttctactcccctcccttctccttatgctgataattcttgttaaatactctgcaggagaggtacaaatttcaacatccacggccaaaaagaccaaagtcactgagaatatatgaatgtcatattggaatgagtagtccggtgggcaccactccttaaatacttatatgtcttacctatttcttatcttctagttgactatatatgatggttatatacatgaaatatctaaattgattattaacttctgataaaatttcaggagcctaaaataaatacatatgcagagtttagagatgatgtacttcctcgtataaagaagcttggctacaatgctctccagataatggccattcaagagcattcttattatgctagctttgggtactttctttttatttatttatttttttggtagtgattctactattgttttgctctacataattgaggagttctctaattctgtcgtcattcattgacaattgttactataactttcaactattattacttttttctaatacaaattcttacccatttacatgattcagctatcatgttacgaatttctttgcaccaagcagtcgttttgggactccagatgatcttaaatctttgattgatagagcccatgaacttggactggttgttctaatggatattgtgcacaggtaataaatggatctttacttcacttcaggaactttctatggtctgtagttgagttcatttcatgtctctgttttatgttcctatacattctagcaaagctcttttgcacaattataataaaaatataaatgatagttagattaacaaatgaaaaagtggagagcagatgaagtgttctagagcttgccccccattatgaatagatgtagtgttctttatgagaatgctagagtcttgttgcaaacaggaaaagaggctgggatgttaaataaggctgctgagttctattagtaggttggaaattacttcatggtgacaagtctgattgaatgaaccttttcattatgttttttgtagaatgagctttgatataatatttggcacaacaaaatttggtagctttggtagtttgaattagaataattttcattgatcaaaagttgctttatagggtatcctaatggatttatttgtttattgtacttttctgatctttcccgatgggtcctgtaatttatttttaaatgctacccttgagttaattttatttggttataagtacataaatcttcttggataaccttttaattttttttatttttttttttttgtgatatgacctattggcaaaaggaagtacattttaacatacatccataattgtctccctgtgcttatattttctgacattctctttttgctttgtatctttattgattgcagtcatgcgtcaaataatactttggatgggctgaacatgtttgatggtacagatagttgttatttccactctggaacccggggttatcattggatgtgggattctcgtctcttcaactatggaaactgggaagtatgtttcttcttgcaaagttgatatggttttcaaagccagtatcattgtatcttctgcaatcagaactagttttttcgcttgtgataggtactgaggtatctgctctcaaatgcaagatggtggttggatgagtacaagtttgatggcttcagatttgatggtgtgacttcaatgatgtatactcatcatgggttgagtgtacgatctaattcatcacttgataaatgcaatgatttgtctgtatttcgtattcctaatgaaaacatgaggatattttactgttattatttttcattccatcctaacattaagctatccgctatgttgactgagtcttttatttttcttaggtaggattcactggaaactacagtgaatattttgggtatgcaactgatgttgatgctgtggtgtatctgatgctcgtcaatgatctcattcatggtctcttccctgaagccataaccattggtgaagatgtatgtattcggtttgagtattaattataattcaactaaatgcttgttgattttgtttggaatttgacatgtaagtgtgatgattcattattcctactaaacctattggaaactgagattgcaaaacctataaaatgatgctggaaggttaatatttagttattacccataatttccaagcccagaaacactcaaaatctcatgaaaactagagtctccttggttgtctgagcgaaaaggactaatagatgatgttatagcacaatagcaagaggaattatgcttccttgcggacaggttgtaaatatatgtttacagaataggccaactatcttgaatatagtttttaaaaaataaagcacattgtttgtgacttttgggcttcttccagaatggttatctttcggctttgtatgttgtagcttttctttgtcattgagaattgaaaggtagatgtagattactaattctgatgatggccagaccttcaattttgcatgaatatctatatgttaattcgtgcataaaattgcagttgattcatgcaataggaatggggatttatcttatgactgatctttagaggacttttactattcaggttagtggcatgccagcattttgtattcccgtcagagatggaggcgtgggttttgactatcggcttcatatggcaattcccgataaatggattgagattctcaagtaagtttatctgtttgtgctctatatgaattgtgagacgggaatgcatgtttgctctcttgtttattatctttccattatgggttaaaagtaatttacattggtgtttcatatcatcaatctgtttgcttaaaatgtctggtacaggagaagagatgaggattggcaaatgggtgaaattgttcacacacttactaacagaagatggctggaaaaatgtgtttcttatgctgaaagtcacgaccaagctcttgttggtgataaaactatagcgttctggttgatggacaaggttgtctaagcttttgttcgtctttgtcatcattcccttgaaacttggcttgaacatgatgttcgaagggataaacttttctattttgcattaatgagcatatatagccattgccgaggatatctgtttatgccttttttggtcagtaagtaggatatactttaatttggtgttggggcacgccattcaatgatttcctccttcttctaggacatgtatgacttcatggctctggatagaccagcaacgccagtaatagaccgtggaatagcattgcataaaatgatcagacttataactatgggattaggaggagaaggatatctaaatttcatggggaacgaatttggtcacccaggtatggatttggatgcttcctatgcatagttcctacttaattccattatacagttttaaccgattggctatttgcatgctttatgtgagaaattatgcaaaatttatttttcagagtggattgatttccctagaggtgaacaacgccttcctgatggttcagttctccctggaaacaatttcagctatgataaatgccgtcgtagatttgatctggttagtattcactgtggcttgtacaaatatttggcattaatacacaataaggtataactgtaaataacataatgtcaaagccttaaaggttgcaatttgctattcagagaagccttttaatttatagctggtagaacgggtgccttgtcatatgatatcacactgtgaattgcggttgagttgtttcttataatccaactttgctcgatatttagggtgatgcagattatctaagatatcggggcatgcaagaatttgaccaagctatgcatcaccttgaagaaaaatatggcgttttctcttgctcgttttaattaatgtggagtaccttttcggatatccttatgctattatttattgtatgaaattgtattaaattcttttaatgtacatttcacggacaagttttctcctgtaaatcattgcagttcatgacggcagaacaccagtacatatcacgccaggacgaaggagatagggtgatcatttttgaaaggggtgacctggtgtttgtcttcaatttccactggacaaatagttattcggattaccgtgtaggctgcttaaagccaggaaaatataaggtacgactgcgaactatcaacatttctttcggtgccatggtaggtgtagagtcatgagtcaggtacggtcactcattcccgcattctattaacatatcgtcatcttccaggtagccctggactccgacagtccgctgtttggaggctttggcagagttcaacccgatgcagaatttttcacattcgtaagatagaaattctcgatatttcttttatctgatcccttgcttgttacgttgcatcttttttgtcacggtccaagaaaaggtatcactacccgtaatttaactgctcgtacaggaagggtaccacgatgatcgtcctcgttccttcatggtgtacgcacccagtagaactgcagtggtgtacgctctagcgaaggaggaggacgaagcaaaaccagtcgaggaatgagcttcgaggtgcactccatggacggtggacgccattttgatgccaacacgcccatcgtcctctcataccaaaaacgcgctccatgttgaaacacagaagtcgccagccatgaacataacttcgcttgccaactctcctctccattctttcattccaaaccggaccaaaaacgcatcagatggagagcgtggtaaggatgaaattaaagctcggataaattagcgaggaagcagtagttagtgtcttctctactctcgtttctatgatttcttgcttttccattctgtaaaatctgctctgcattttctgtattgccataacttcttgacatgtatttcccagcagataatatttgttggctataatgctatgattaagaggaaaataaaaactgcatttcattgtccatctttcattccctgatgccctttaataaatgaatagagcctaaatatttgtatgctcgtttgctaagagctcgtgaaagtattcgttagtgttcttttaataatgttcataaacaagttcgtttagtgtttgtttaataatgtttacgaatatatgttcgtttaagat
