# Supplementary material for: CRISPR/Cas9-Based Mutagenesis of Starch Biosynthetic Genes in Sweet Potato (Ipomoea Batatas) for the Improvement of Starch Quality
Source: Int J Mol Sci. 2019 Sep 23;20(19):4702. doi: 10.3390/ijms20194702 (PMC6801948; doi:10.3390/ijms20194702)
Supplement: Supplementary file 1 [file ijms-20-04702-s001.zip › ijms-583672-for proofreading sup/supplementary Table 2 PCR primer for resubmission 9 4 2019.doc]

| Gene name | primer | site in genome（ATG+1） |
| --- | --- | --- |
| GBSSI-1F | TAGTGGCCTAGTGCATGTCAAATC | -234bp |
| GBSSI-1R | CGTTCATGTAGATTCCTCTCGACTG | +652bp |
| GBSSI-2F | CAATCTCTTTGATGGGTTGTTTTC | -182bp |
| GBSSI-2R | GCAACAACAAAACTTCGTTACCTCT | +2030bp |
| SBEII-1F | TTGGCTACAATGCTCTCCAGATAAT | +5660bp |
| SBEII-2R | GTGACAAAAAAGATGCAACGTAACG | +9730bp |
| SBEII-2F | TTGTCAGCATTCCCTTGAAACTTAG | +8367bp |
| SBEII-2R | GTGACAAAAAAGATGCAACGTAACG | +9730bp |

Table S2. Primers for the target genes for sequence
